# Post-Liver Transplant Kidney Dysfunction: Incidence of Acute Kidney Injury and Chronic Kidney Disease and Risk Factors Related to Chronic Kidney Disease Development

**DOI:** 10.3390/diseases13050144

**Published:** 2025-05-06

**Authors:** Ana Flavia Moura, José A. Moura-Neto, Beatriz de Melo Ribeiro, Paula Ribeiro Oliveira, Arthur Guimarães de Freitas, Alessandra Lima Costa, Daniela Moura-Landim, Liana Codes, Paulo Lisboa Bittencourt, Constança Margarida Sampaio Cruz

**Affiliations:** 1 Bahiana School of Medicine and Public Health, Salvador 40285-001, BA, Brazil; jamouraneto@hotmail.com (J.A.M.-N.); bia.meloribeiro1909@gmail.com (B.d.M.R.); paularibeiro20.2@bahiana.edu.br (P.R.O.); arthurfreitas21.1@bahiana.edu.br (A.G.d.F.); alessandracosta21.1@bahiana.edu.br (A.L.C.); danielaqmoura@hotmail.com (D.M.-L.); lianafoulon@bahiana.edu.br (L.C.); paulobittencourt@bahiana.edu.br (P.L.B.); constancacruz@yahoo.com.br (C.M.S.C.); 2 Portuguese Hospital, Salvador 40130-030, BA, Brazil; 3 Santo Antônio Hospital—OSID, Salvador 40415-180, BA, Brazil

**Keywords:** liver transplant, acute kidney injury, chronic kidney disease, renal replacement therapy, postoperative complications

## Abstract

**Introduction:** Acute kidney injury (AKI) and chronic kidney disease (CKD) are common complications following liver transplantation (LT), significantly impacting graft and patient survival. AKI affects more than 50% of LT recipients, with a subset requiring renal replacement therapy (RRT), while CKD develops in up to 60% of cases, increasing long-term morbidity and mortality. This study aimed to determine the incidence of AKI and CKD post-LT and to identify risk factors associated with CKD development. **Methods:** All adult cirrhotic patients without concurrent CKD submitted to LT between January 2001 and December 2017 at the main transplant center in Salvador, Bahia, Brazil, with more than 6-month survival were included in the study. AKI was defined by KDIGO criteria within the first 7 days post-LT. CKD was diagnosed in the presence of the estimated glomerular filtration rate (eGFR) < 60 mL/min/1.73 m^2^ and/or proteinuria ≥ 200 mg/g 1 and 5 years after LT. Clinical and biochemical parameters were analyzed using multivariate logistic regression to identify independent predictors of CKD. **Results:** A total of 177 LT recipients (72.9% male; mean age 53.6 ± 12.6 years) were studied. AKI occurred in 51.4% of them in the first 7 days after LT, and 10 (11%) required RRT. CKD was diagnosed in 30% of LT recipients at 1 year and in 42.9% at 5 years. The majority displayed CKD stage G3 (72.4% at 5 years). Multivariate analysis identified pre-LT serum creatinine (OR 7.74, 95% CI 1.99–30.02) and AKI within 7 days after LT (OR 2.72, 95% CI 1.22–6.06) as independent predictors of CKD development. **Conclusions:** AKI is highly prevalent in the early post-LT period and is a major determinant of CKD progression. Pre-LT renal function and perioperative AKI were significantly associated with worse long-term nephrological outcomes. Optimized perioperative management and renal monitoring strategies are crucial to minimize progression to end-stage kidney disease in LT recipients.

## 1. Introduction

Liver transplantation (LT) is the primary treatment for patients with end-stage liver disease (ESLD) [1]. Acute kidney injury (AKI) is a common complication in the postoperative period of LT, driven by several factors, including the increasing prevalence of AKI and hepatorenal syndrome in patients with ESLD on the LT waiting list, the physiological impact of surgery, systemic inflammation, perioperative hemodynamic instability, and the use of nephrotoxic agents, particularly calcineurin inhibitors (CNIs). Several studies have reported AKI in more than 50% of LT recipients and AKI requiring renal replacement therapy (RRT) in approximately 15% of cases within the first one to two weeks post-transplant [1,2,3,4]. A recent meta-analysis, which included 30 large-scale studies encompassing 13,653 post-LT patients, reported an overall AKI prevalence of 46.3%, with incidence rates ranging from 21.8% to 87.2% [5]. Additionally, the pooled RRT frequency in these studies was 9.5%, with reported rates varying between 4.0% and 36.2% [6].

Several studies have highlighted the impact of AKI in the post-LT period [5]. It is well established that patients who develop AKI in the early days following LT experience worse outcomes, including higher mortality rates, prolonged hospitalization, and an increased risk of acute rejection [7,8,9,10,11]. While some studies have demonstrated a higher long-term incidence of chronic kidney disease (CKD) in LT recipients who experienced AKI in the immediate postoperative period, this association has not been consistently reported across all studies [7,8,11].

The incidence of CKD among patients who experience AKI in the early post-LT period ranges from 15% to 60%, depending on CKD stage and AKI severity [10,11]. Additionally, other risk factors, including post-LT type 2 diabetes mellitus (DM) and hypertension (HTN), the use of CNIs, and preoperative MELD scores, have also been associated with an increased risk of CKD development following LT [7,8,9,10,11].

While the estimated global prevalence of CKD ranges between 10% and 13%, a European retrospective study reported a CKD incidence of 32% in LT recipients one year post-transplant, increasing to 37% after two years [12]. Cullaro et al. found that LT recipients who developed CKD had a 16% higher mortality rate compared to those without CKD [13]. Moreover, for every 90 days with a CKD diagnosis, the mortality rate increased by 2.7% in this population [13].

In general, the most frequently observed stages of CKD post-LT are G2 and G3, accounting for approximately 30% to 60% of cases [10,11]. The prevalence of more advanced stages (G4 and G5) varies between 15% and 25%, depending on the post-LT follow-up period [8,9]. CKD progression at any stage following LT is independently associated with increased mortality and a higher risk of cardiovascular events [3,11], significantly impacting healthcare costs and patients’ quality of life.

Although the survival of LT recipients has improved in recent decades, implementing strategies for the prevention and early diagnosis of renal diseases, including both AKI and CKD, can significantly reduce the financial, clinical, and social burdens associated with their management in this population. This study aimed to assess the incidence of AKI within the first 7 days post-transplant, the prevalence of CKD more than one year after LT, and the factors associated with CKD development in these patients.

## 2. Methods

This is an ambispective observational study including patients over 18 years old who underwent LT between January 2001 and December 2017 at the main transplant center in Salvador, Bahia, Brazil. Patients with pre-existing CKD before LT, those undergoing split liver transplantation or retransplantation, and those who died within the first 5 years post-LT were excluded. No patients with elevated serum creatinine at the admission for the LT were included. All patients in the sample had normal renal function at baseline, as evidenced by an estimated glomerular filtration rate (eGFR) greater than 60 mL/min, calculated using admission serum creatinine and considering weight, height, and age. It is important to clarify that all patients included in this study had enough data 1 year and 5 years post-LT to diagnose CKD. Given its ambispective design, the study was divided into two parts, as follows:

Part 1: Retrospective Observational Study

A portion of LT patients from the Portuguese Hospital (HP) treated between January 2001 and December 2017 was retrospectively analyzed using the institution’s liver transplant database. Data were initially collected from an anonymized database. Missing data were retrieved from the Track and Tasy electronic medical record system and recorded in an Excel spreadsheet between January 2023 and June 2024.

Part 2: Prospective Observational Study

LT patients between January 2001 and December 2017 who continued outpatient follow-up at HP and agreed to participate were prospectively investigated for CKD through the measurement of their urine protein/creatinine ratio (UPCR) in urine samples, as well as their serum creatinine levels. UPCR was included in routine postoperative laboratory tests without interfering with patient follow-up, while serum creatinine was already part of the long-term laboratory assessments conducted at least semiannually. Therefore, no patient underwent a UPCR measurement at the time of liver transplant admission or during the in-hospital period, as this test was only incorporated into the outpatient follow-up routine for these patients after the year 2022. All patients included in the sample were under follow-up at both 1 year and 5 years after liver transplantation. All were enrolled in the prospective phase and underwent UPCR collection, except for those who already had a diagnosis of CKD G4 or G5. Patients who died within 5 years post-transplant were excluded from the study.

### 2.1. Variables of Interest

Demographic Data: Age and gender.

Pre-LT Recipient Data: Weight; height; MELD score at admission for the LV; cause of liver failure, i.e., autoimmune hepatitis, non-alcoholic steatohepatitis (NASH), hepatitis B, hepatitis C, cryptogenic cirrhosis, alcoholic liver disease (ALD) and others; comorbidities (overweight/obesity, DM, HTN, and dyslipidemia); baseline serum creatinine (measured at admission for the LT); and baseline eGFR (measured using baseline serum creatinine).

Post-LT Recipient Data: Serum creatinine in the first 7 days post-LT, quarterly in the first year post-LT, and annually from the second to the fifth year post-LT; UPCR quarterly in the prospective study phase; need for RRT (yes/no).

### 2.2. Outcome Definition Criteria

These criteria included the occurrence of AKI in the first 7 days of the TH and development of CKD in the postoperative follow-up. The diagnoses of AKI and CKD were made based on the criteria recommended by the Kidney Disease Improving Global Outcomes (KDIGO) initiative [14], considering the following definitions:

AKI: Increase in serum creatinine ≥0.3 mg/dL within 48 h or increase to ≥1.5 times baseline within the past 7 days.

CKD: Functional (eGFR < 60 mL/min/1.73 m^2^) or structural renal abnormalities (UPCR ≥ 200 mg/g) persisting for ≥3 months, with health implications.

For the definition of these outcomes, parameters such as urinary output for AKI diagnosis, as well as findings from imaging studies or kidney biopsy for CKD diagnosis, were not analyzed.

### 2.3. Statistical Analysis

Data were entered into an SPSS 11.0 database for statistical analysis. Categorical variables were expressed as valid percentages and absolute values, while continuous variables were presented as means and standard deviations (SD) or medians (IQR), depending on distribution. Distribution was assessed using the Kolmogorov–Smirnov and Shapiro–Wilk tests. The Chi-squared test was used for proportion comparisons, and Fisher’s exact test was applied, when appropriate. Multivariate analysis employed logistic regression for variables with *p* < 0.20 in univariate analysis; *p* < 0.05 was considered statistically significant.

### 2.4. Ethical Considerations

This research was approved by the Ethics Committee of Português Hospital (CAAE 65841322.1.0000.5029). Retrospective data collection (Part 1) was exempt from informed consent. All prospective participants (Part 2) signed informed consent forms.

## 3. Results

**Figure 1:**
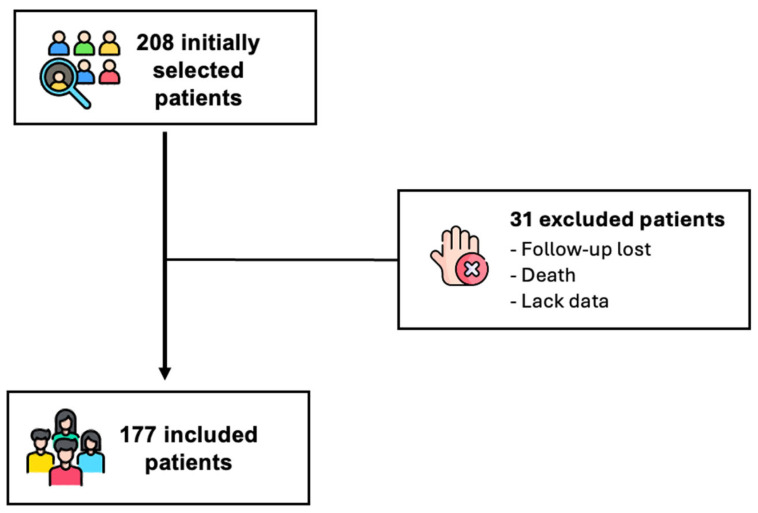
Flowchart of patient selection for the studied sample.

Initially, 208 patients were selected, of whom 31 were excluded either due to refusal to participate, loss to follow-up, or a lack of clinically relevant data. The patient selection flowchart is presented in Figure 1.

Demographic, clinical, and laboratory data for those remaining 177 patients are depicted in Table 1. Briefly, 129 (72.9%) were male, and the mean age was 53.61 years (±12.65). Regarding comorbidities, 51.1% and 52.9% exhibited, respectively, T2D and HTN. Less than half of the patients were overweight or obese (32.6%), while 23.7% displayed dyslipidemia. The most common indications for LT were viral cirrhosis due to chronic viral hepatitis and ALD (Table 1). All patients received triple-based immunosuppression with calcineurin inhibitors (CNIs), either tacrolimus (n = 175) or cyclosporin (n = 2), in association with corticosteroids and mycophenolate in the first 3 and 12 months after LT, respectively.

The incidence of AKI in the first 7 days post-LT was 51.4% (91 patients). Regarding AKI severity, most cases were classified as KDIGO 1 (62.6%). Of the 91 patients who developed AKI, 10 (11%) required RRT. AKI-related outcome data are shown in Table 2.

Among the 177 patients studied, 30% (53 patients) developed CKD in the first year post-LT. Of these, 19 patients (35.85%) displayed a preserved estimated glomerular filtration rate (eGFR > 60 mL/min/1.73 m^2^) with only structural alterations, i.e., UPCR >200 mg/g. If CKD was defined solely by eGFR reduction to <60 mL/min/1.73 m^2^, the CKD frequency at one year would decrease to 19.2%. Of the 53 patients with CKD one year post-LT, more than half (56.6%) were classified as stage G3, with 32.1% in stage G3B. Only two patients (3.8%) were in stage G4, and two were in stage G5.

After 5 years post-LT, 76 patients (42.9%) had developed CKD. Of these, 13 (17.1%) had an eGFR > 60 mL/min/1.73 m^2^. Excluding UPCR, the CKD frequency 5 years post-transplant would be 35.6% (63 patients). Most patients (64.4%) with CKD 5 years post-LT were in stage G3, with 26.3% (23 patients) in stage G3B. Five patients (6.6%) were in stage G4, and seven in stage G5. CKD frequencies at 1 and 5 years post-LT are detailed in Table 2.

Of all patients in the cohort, nine underwent renal replacement therapy more than 5 years following liver transplantation, including seven on dialysis and two who received a kidney transplant.

A total of 110 patients underwent at least two measurements of proteinuria to assess structural kidney injury. The mean UPCR during the study period was 410 mg/g (SD ± 1080 mg/g). Among those who underwent UPCR testing, 70% (77 patients) displayed proteinuria equal to or below 200 mg/g, 21 patients (19%) had a UPCR between 200 and 500 mg/g, 6 patients (5.5%) had proteinuria between 500 and 1000 mg/g, and 6 patients (5.5%) had values above 1000 mg/g. Patients with pre-existing CKD stages G4 or G5 were not subjected to UPCR testing.

Of the 91 patients who developed AKI in the early post-LT period, 41 (45%) progressed to CKD within the first 5 years post-transplant, with 58.5% receiving a CKD diagnosis within the first year. Among these 41 patients who experienced early postoperative AKI and subsequently developed CKD, 73.2% had been classified as KDIGO stage 1, while 9.8% were classified as KDIGO stage 3 (Table 3).

Among the 177 patients included in the study, 29 (16.4%) died at some point during the study period (January 2001 to December 2017). Of these 29 patients, 16 had experienced AKI within the first 7 days post-transplant, and 13 developed CKD within the first 5 years following liver transplantation, 6 of whom required maintenance dialysis (Table 4).

Age (*p* = 0.003), creatinine level at admission (*p* = 0.000), diagnosis of HTN (*p* = 0.038), and history of AKI in the first 7 days PO (*p* = 0.003) showed statistically significant association with CKD. A total of 78.6% of patients with CKD were male. Overweight/obesity and diagnosis of DM were not associated with the development of CKD. Among patients with DM (n = 89), 36% (32 patients) progressed to CKD (*p* = 0.276). Among patients with CKD, 68.5% did not have overweight/obesity (*p* = 0.838). Table 5 summarizes the factors associated with the development of CKD in the post-TH period.

The multivariate analysis identified admission creatinine (OR 7.74 [1.99–30.02]) and AKI history within 7 days post-LT (OR 2.72 [1.22–6.06]) as independent predictors of CKD (Table 6).

## 4. Discussion

This study included 177 LT recipients with no prior diagnosis of CKD. Patients were selected from medical records and outpatient follow-ups at a private center in Salvador, Bahia, Brazil, which accounts for over 90% of all LTs performed in the state [15]. This highlights the representativeness of the selected sample in reflecting the local LT recipient population. Epidemiological studies on LT patients commonly report demographic characteristics similar to those observed in our cohort, with more than half of the patients being male and a mean age exceeding 45 years [16,17,18], a pattern consistent with our findings.

In this cohort, the most common etiology of liver failure was viral disease, particularly hepatitis C virus (HCV) infection. This finding contrasts with more recent data indicating that viral infections are becoming an increasingly rare indication for LT due to the availability of effective treatments. The high prevalence of HCV- or HBV-related liver failure in our study is likely explained by the inclusion of patients who underwent transplantation more than a decade ago, before the widespread implementation of curative antiviral therapies.

The incidence of post-LT AKI is reported to be high, typically exceeding 50% [1,3], which is consistent with the incidence observed in this study (51.4%). Early post-LT AKI is associated with adverse outcomes, including increased mortality, prolonged hospitalization, a higher risk of graft loss, and a greater likelihood of progression to CKD [8,14,15,16,17,18]. These complications place a significant burden on healthcare systems and negatively impact patient survival and quality of life, underscoring the critical need for strategies aimed at preventing early postoperative AKI [19,20].

The severity of AKI is a key determinant of the risk of developing comorbidities, particularly CKD. In this study, most patients who developed AKI within the first 7 days post-LT were classified as KDIGO 1, representing the mildest stage of AKI. Additionally, 11% of patients required RRT, a frequency comparable to the approximately 15% reported in the literature [3,4].

The prevalence of CKD within the first year post-LT has been reported to range from 4% to 28% [20]. In this study, 30% of patients developed CKD one year after LT, a notably high prevalence. Kang et al. suggested that the most significant decline in estimated eGFR occurs within the first year post-transplant [21]. An important observation is that many studies assessing CKD prevalence at different post-LT stages focus solely on functional renal impairment, excluding structural damage [22,23]. As demonstrated in our findings, omitting structural damage from the CKD diagnosis reduced its prevalence from 30% to 19.2%. This finding may serve as a warning regarding the importance of improving renal function assessment in the follow-up of patients undergoing LT. It highlights the need to include UPCR testing in the post-transplant follow-up of LT recipients in order to enable early diagnosis of CKD and the implementation of strategies aimed at slowing disease progression, even before a significant decline in eGFR is observed.

Consistent with previous studies [22,23], the majority of CKD patients one year post-LT were classified as stage 3 (60.4%), with few or no cases progressing to advanced stages (G4 or G5)22. The low prevalence of advanced CKD stages within the first year post-LT in patients without preexisting CKD is expected, given the typical progression timeline of the disease. Early and effective management of CKD in its initial stages is essential to preventing its progression to more severe forms, which often require long-term renal replacement therapy (RRT).

Five years post-LT, nearly 43% of patients in this cohort had developed CKD, predominantly at stage G3. The progressive increase in CKD prevalence over time among transplant recipients is well-documented [16,24]. A key factor contributing to this trend is the use of CNIs, the primary immunosuppressive agents for most liver transplant recipients [10,22]. CNIs have well-known nephrotoxic effects, necessitating careful monitoring of serum levels to balance the risk of graft rejection with the potential for kidney damage. It is important to note that all patients in this study received CNIs as part of their immunosuppressive regimen; therefore, immunosuppression could not be considered a variable associated with CKD development in our analysis.

Among the 91 patients who developed AKI within the first 7 days post- LT, 45% progressed to CKD within the first 5 years post-LT. Of these 41 patients, 58.5% were diagnosed with CKD within the first year post-transplant. The high prevalence of CKD among patients with a history of AKI is well-documented in the literature, and our findings further reinforce that AKI is an independent risk factor for CKD development.

Other factors frequently associated with CKD development post-LT include pre-existing DM, the need for RRT, elevated MELD score at admission, older age, female sex, and HTN, with some variation across studies [13,14,22]. A meta-analysis of 21 studies, encompassing 44,383 patients, identified age, female sex, and early post-LT AKI as significant risk factors for CKD [23]. In our study, age (*p* = 0.003) and early post-LT AKI (*p* = 0.003) were also significantly associated with CKD development. However, most CKD patients in our cohort were male (78.6%), and DM was not found to be a significant risk factor for CKD (*p* = 0.276). Additionally, admission serum creatinine levels (*p* = 0.000) and HTN diagnosis (*p* = 0.038) were both associated with an increased risk of CKD. Differences in sample size and the clinical demographic characteristics of the study populations may account for these variations in post-LT CKD risk factors. In recent decades, there has been significant progress in the available strategies for the conservative management of CKD, and the selection and appropriate use of these strategies—including RAAS inhibitors, SGLT2 inhibitors, mineralocorticoid receptor antagonists, and GLP-1 receptor agonists—may influence and potentially modify the course of the disease. Notably, immunosuppressive regimens were not analyzed as variables in this study.

Multivariate analysis identified only serum creatinine at admission for transplant—OR 7.74 (1.99–30.02)—and a history of AKI—OR 2.72 (1.22–6.06)—as significant predictors of CKD. These two factors have been consistently associated with CKD in previous studies, with AKI recognized as an independent risk factor for CKD, even in non-transplant populations [4,8,9,10,11]. It is worth noting that serum creatinine levels at admission were within the normal range for all patients included in this study. This finding may suggest that patients with liver disease, who generally have a lower percentage of lean body mass, require closer monitoring of renal function, even when creatinine values appear to be within normal limits.

Among the 177 patients included in the study, 29 (16.4%) died at some point during the study period (January 2001 to December 2017). It is important to note that all deaths occurred more than 5 years after liver transplantation, as patients who died before this time point were excluded from the analysis. Of these 29 patients, 16 had experienced AKI within the first 7 days post-transplant, and 13 developed CKD within the first 5 years following liver transplantation, 6 of whom required maintenance dialysis.

This study has some limitations that should be considered when interpreting its results. As it was conducted in a single hospital in Brazil, the findings may not be fully generalizable to other centers or populations in different regions. However, this limitation is somewhat mitigated by the fact that this institution is the primary transplant center in the state, performing over 90% of all liver transplants in the region. Another potential limitation is the reliance on medical record data, collected both retrospectively and prospectively, which may introduce documentation errors, particularly in the retrospective period, potentially affecting the accuracy of the analyzed variables. Lastly, the uniform use of CNIs as the immunosuppressive regimen for all transplant recipients prevented the inclusion of immunosuppression as a variable in this study, which may have influenced the occurrence of renal dysfunction. Another potential factor influencing the progression to CKD and its advancement to more severe stages is the use of pharmacological strategies for conservative CKD management, which was not controlled for in this study.

Despite some limitations, this study presents findings that align with the results in the literature. AKI and CKD are common complications following liver LT, and a better understanding of their prevalence and risk factors is essential for developing effective strategies for prevention, early detection, and optimal management.

## 5. Conclusions

The high incidence of renal dysfunction underscores the importance of ongoing renal health monitoring in LT recipients. Routine evaluation of these patients should include screening for kidney injury through serum creatinine measurement and urine protein-to-creatinine ratio assessment to enable early detection and intervention.

## Figures and Tables

**Table 1:** Clinical and demographic data for the patients (n = 177).

Variables *	n (%)
**Age (years)**	53.61 ± 12.65
**MELD score at admission**	19.87 ± 8.16
**Creatinine at admission (mg/dL)**	0.83 ± 0.30
**eGFR at admission (mL/min/1.73 m^2^)**	99.90 ± 15.42
**Gender**	
Female	48 (27.1)
Male	129 (72.9)
**Diabetes Mellitus**	
No	85 (48.9)
Yes	89 (51.1)
**Hypertension**	
No	82 (47.1)
Yes	92 (52.9)
**Dyslipidemia**	
No	132 (76.3)
Yes	41 (23.7)
**Overweight/Obesity**	
No	116 (67.4)
Yes	56 (32.6)
**Indications for liver transplantation**	
Cryptogenic cirrhosis	13 (7.3)
Autoimune hepatitis	11 (6.2)
NASH	10 (5.6)
Other causes	71 (40.1)
ALD	45 (25.4)
HBV	9 (5.1)
HCV	63 (35.6)

* Quantitative variables expressed as mean and standard deviation. Qualitative variables expressed as absolute number and valid percentages. NASH: non-alcoholic steatohepatitis; HBV: hepatitis B virus; HCV: hepatitis C virus; ALD: alcoholic liver disease; eGFR: estimated glomerular filtration rate.

**Table 2:** Frequency of renal dysfunction and rate of patients requiring RRT in the first 7 days after liver transplantation.

Variables *	N (%)
**AKI < 7 days PO (n = 177)**	
No	86 (48.6)
Yes	91 (51.4)
**AKI stage < 7 days PO (n = 91)**	
KDIGO 1	57 (62.6)
KDIGO 2	17 (18.7)
KDIGO 3	17 (18.7)
**RRT < 7 days PO (n = 91)**	
No	81 (89)
Yes	10 (11)
**CKD after 1 year PO (n = 177)**	
No	124 (70)
Yes	53 (30)
**CKD stage after 1 year PO (n = 53)**	
G1	7 (13.2)
G2	12 (22.6)
G3A	13 (24.5)
G3B	17 (32.1)
G4	2 (3.8)
G5	2 (3.8)
**CKD after 5 years PO (n = 177)**	
No	101 (57.1)
Yes	76 (42.9)
**CKD stage after 5 years PO (n = 76)**	
G1	7 (9.2)
G2	8 (10.5)
G3A	39 (38.1)
G3B	20 (26.3)
G4	5 (6.6)
G5	7 (9.3)

* Quantitative variables expressed as mean and standard deviation. Qualitative variables expressed as absolute number and valid percentages. CKD: chronic kidney disease; AKI: acute kidney injury; RRT: renal replacement therapy; PO: postoperative.

**Table 3:** Number of patients with AKI in early post-LT that developed CKD in the first 5 years post-transplant.

Variables *	N (%)
**AKI < 7 days PO (n = 177)**	
No	86 (48.6)
Yes	91 (51.4)
**CKD in the first year PO (n = 91)**	
No	67 (73.6)
Yes	24 (26.4)
**CKD after 5 years PO (n = 91)**	
No	50 (55)
Yes	41 (45)

* Quantitative variables expressed as mean and standard deviation. Qualitative variables expressed as absolute number and valid percentages. CKD: chronic kidney disease; AKI: acute kidney injury; LT: liver transplant.

**Table 4:** Number of deaths occurring beyond 5 years after liver transplantation.

Variables	N (%)
**Death**	**29 (16.4)**
Without AKI < 7 days or CKD < 5 years PO	10 (34.5)
AKI < 7 days PO	16 (55.2)
CKD < 5 years PO	13 (44.8)
CKD and RRT < 5 years PO	6 (20.7)

AKI: acute kidney injury; PO: post-operative; CKD: chronic kidney disease; RRT: renal replacement therapy.

**Table 5:** Factors associated with the development of CKD in the post-TH period (n = 177).

Variables *	Without CKD n (%)	With CKD n (%)	*p*-Value **
**Age (years)**	51.47 ± 13.75	58.16 ± 5.33	0.003
**MELD score at admission**	20.46 ± 8.85	18.79 ± 6.70	0.314
**Creatinine at admission (mg/dL)**	0.76 ± 0.26	0.97 ± 0.34	0.000
**eGFR at admission (mL/min/1.73 m^2^)**	100.96 ± 14.90	90.79 ± 14.91	0.000
**Gender**			0.247
Female	36 (29.8)	12 (21.4)	
Male	85 (70.2)	44 (78.6)	
**Diabetes Mellitus**			0.276
No	61 (51.7)	24 (42.99)	
Yes	57 (48.3)	32 (57.1)	
**Hypertension**			0.038
No	62 (52.5)	20 (35.7)	
Yes	56 (47.5)	36 (64.3)	
**Dyslipidemia**			0.255
No	93 (78.8)	39 (70.9)	
Yes	25 (21.2)	16 (29.1)	
**Overweight/obesity**			0.838
No	79 (66.9)	37 (68.5)	
Yes	39 (33.1)	17 (31.5)	
**AKI < 7 days PO**			0.003
No	68 (56.2)	18 (32.1)	
Yes	53 (43.8)	38 (67.9)	
**AKI stage < 7 days**			0.011
No	68 (56.2)	18 (32.1)	
KDIGO 1	30 (24.8)	27 (48.3)	
KDIGO 2	12 (9.9)	5 (8.9)	
KDIGO 3	11 (9.1)	6 (10.7)	
**RRT < 7 days PO**			0.180 ^&^
No	112 (95.7)	50 (90.9)	
Yes	5 (4.3)	5 (9.1)	
**Cause of Liver Failure**			0.419
Cryptogenic cirrhosis	9 (7.4)	4 (7.1)	
Autoimune Hepatitis	8 (6.6)	3 (5.4)	
NASH	5 (4.1)	5 (8.9)	
Others	20 (16.5)	6 (10.8)	
ALD	34 (28.2)	11 (19.6)	
HBV	5 (4.1)	4 (7.1)	
HCV	40 (33.1)	23 (41.1)	

* Quantitative variables expressed as mean and standard deviation. Qualitative variables expressed as absolute number and valid percentages ** Chi-squared test; ^&^ Fisher’s Exact Test. CKD: chronic kidney disease; AKI: acute kidney injury; RRT: renal replacement therapy; PO: postoperative; NASH: non-alcoholic steatohepatitis; HBV: hepatitis B virus; HCV: hepatitis C virus; ALD: alcoholic liver disease; eGFR: estimated glomerular filtration rate.

**Table 6:** Predictive factors for CKD in the post-liver transplant period.

Preditors	Crude Odds	Adjusted Odds
**AKI < 7 days after LT**	2.71 (1.39–5.27)	2.72 (1.22–6.06)
**Creatinine at admission**	-	7.74 (1.99–30.02)

AKI: acute kidney injury.

## Data Availability

Data available on request due to restrictions.
